# Interpretable time-aware and co-occurrence-aware network for medical prediction

**DOI:** 10.1186/s12911-021-01662-z

**Published:** 2021-11-02

**Authors:** Chenxi Sun, Hongna Dui, Hongyan Li

**Affiliations:** 1 School of Electronics Engineering and Computer Science, Peking University, No. 5 Yiheyuan Road, Beijing, 100871 People’s Republic of China; 2 Key Laboratory of Machine Perception (Ministry of Education), Peking University, Beijing, 100871 People’s Republic of China; 3 The Aviation Industry Corporation of China, Ltd, Chengdu Aircraft Design & Research Institute, Chengdu, 610041 People’s Republic of China

**Keywords:** Medical prediction, Interpretable deep learning, Electronic health records, Disease correlation

## Abstract

**Background:**

Disease prediction based on electronic health records (EHRs) is essential for personalized healthcare. But it’s hard due to the special data structure and the interpretability requirement of methods. The structure of EHR is hierarchical: each patient has a sequence of admissions, and each admission has some co-occurrence diagnoses. However, the existing methods only partially model these characteristics and lack the interpretation for non-specialists.

**Methods:**

This work proposes a time-aware and co-occurrence-aware deep learning network (TCoN), which is not only suitable for EHR data structure but also interpretable: the co-occurrence-aware self-attention (CS-attention) mechanism and time-aware gated recurrent unit (T-GRU) can model multilevel relations; the interpretation path and the diagnosis graph can make the result interpretable.

**Results:**

The method is tested on a real-world dataset for mortality prediction, readmission prediction, disease prediction, and next diagnoses prediction. Experimental results show that TCoN is better than baselines with 2.01% higher accuracy. Meanwhile, the method can give the interpretation of causal relationships and the diagnosis graph of each patient.

**Conclusions:**

This work proposes a novel model—TCoN. It is an interpretable and effective deep learning method, that can model the hierarchical medical structure and predict medical events. The experiments show that it outperforms all state-of-the-art methods. Future work can apply the graph embedding technology based on more knowledge data such as doctor notes.

## Background

**Fig. 1:**
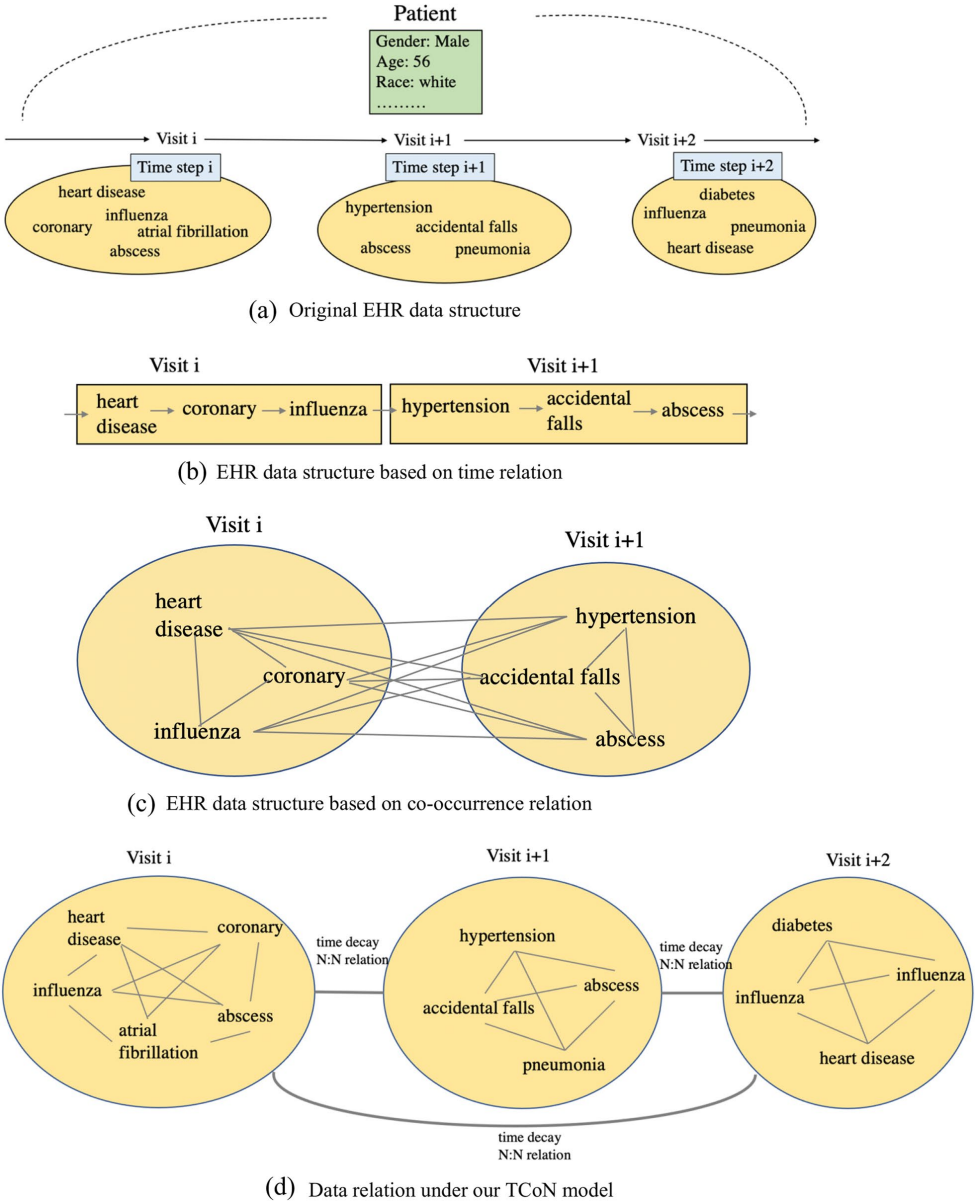
The data structure of EHR based on different methods. **a** Original EHR data structure. **b** EHR data structure based on time relation. **c** EHR data structure based on co-occurrence relation. **d** Data relation under our TCoN model. The data form **b** arranges codes in a random order, but different sequences have different effects on results. For example, the sequence ‘heart disease —> influenza —> coronary’ has closer relation between ‘heart disease’ and ‘influenza’ than the sequence ‘heart disease —> coronary —> influenza’. The data form **c** can make every two codes have the equal relation, but if ‘heart disease’, ‘atrial fibrillation’ and ‘diabetes’ are in three different visits, the equal relation will fail as there are different time intervals among them. The data form **d** is the combination. it describes both the equal code relation in the same visit and the time relation in different visits

Electronic Health Records (EHRs) are increasingly popular and widely used in hospitals for better healthcare management. A typical EHR dataset consists of much patient information, including demographic information and medical information. The medical information is an irregular hierarchical patient-visit-code (patient-admission-diagnosis) form, shown in Fig. 1a: (1) Each patient has many visit records as he/she may go to see a doctor many times. The visit records have corresponding time stamps and form a sequence; (2) Each visit contains many codes, which are usually disease diagnoses. The codes have the co-occurrence relation without order. For example, in a patient record, the chronic kidney disease is recorded after a cold record, but we can't conclude that the patient didn't have chronic kidney disease before he caught a cold. Two diagnoses have an uncertain time relation. We call such issues as the co-occurrence relation, such as complication, causation, and continuity. Thus, EHR has both the time relation and the co-occurrence relation.

Medical tasks such as disease prediction [1–3], concept representation [4, 5], and patient typing [6–8] are essential for personalized healthcare and medical research. Nevertheless, the tasks are challenging for physicians, considering the complex patient states, the amount of diagnosis, and the real-time requirement. Thus, a data-driven approach by learning from large accessible EHRs is the desiderata.

In recent years, the Deep Learning (DL) model has made remarkable achievements due to its strong learning ability and flexible architecture [9–13]: some DL methods can model the sequential time relation of medical data. For example, RETAIN [3] utilizes gated recurrent unit (GRU) [14, 15] to predict medical events, Dipole [1] uses Bidirectional RNN (BRNN) [16] to integrate the information in the past and the feature, and T-LSTM [8, 17] injects the time decay effect to handle irregular time intervals. Using these methods, the EHR structure is modeled as Fig. 1b; Some DL methods can model the co-occurrence relation of medical data. For example, Word2Vec [18, 19], Med2Vec [4], and MiME [5] model the medical relations to better express the original data by the idea of representation learning [20–23]. Using these methods, the EHR structure is modeled as Fig. 1c.

However, no method can model both relations simultaneously. Because t there is a conflict between the two relations: The time relation makes data distributed longitudinally but the co-occurrence relation makes data distributed bipartite graph-like. If considering both these two relations, the EHR structure is shown in Fig. 1d.

Meanwhile, in the real-world application, the data-driven method is required to be interpretable to facilitate the use of doctors [24–26]. However, the DL method is the black-box model which is troubled by poor interpretability [27–32].

To address the above issues, in this work, we define EHR as the hierarchical co-occurrence sequence and propose a novel model called Time-aware and Co-occurrence-aware Network (TCoN). TCoN can not only model the two relations simultaneously but also has the ability of interpretation. TCoN has the pre-train and fine-tune mechanism for the imbalanced data and is more accurate than all baselines in medical prediction tasks.

## Materials and methods

In this section, we first introduce the MIMIC-III dataset and the data preprocessing process. Then, we describe the proposed methods in detail.

### Dataset description and preprocessing

MIMIC-III is a freely accessible de-identified medical dataset, developed and maintained by the Massachusetts Institute of Technology Laboratory for Computational Physiology [33]. Based on MIMIC-III dataset, we selectively extract data and form three data sets:

#### Overall dataset

We extract records with more than one visit from MIMIC-III. The new dataset comprises 19,993 hospital admissions of 7537 patients and 260,326 diagnoses with 4,893 unique codes defined by the International Classification of Diseases-9 version (ICD-9). For one patient, the visit number is 2.66 on average. For one visit, the code number is 13.02 on average and up to 39.

#### Sepsis dataset

Following the latest sepsis 3.0 definition [34], we extract 1232 sepsis patients whose SOFA is greater than or equal to 2.

#### Heart failure dataset

According to ICD-9 code, we extract 1608 heart failure patients who have diagnoses of 428.x code.

In sepsis dataset and heart failure dataset, the extracted data is the records for the first time that these two diagnoses appear. And these two datasets are imbalanced. The detailed statistic is shown in Table 1.

**Table 1:** Statics of extracted MIMIC-III dataset

Sepsis codes	SOFA $\ge$ 2
Heart failure codes	428.x
Avg. rate of in-hospital mortality	12.58% (5854/46,520)
Avg. rate of readmission to ICU	16.20% (7537/46,520)
Avg. rate of sepsis	6.16% (1232/19,993)
Avg. rate of heart failure	8.04% (1608/19,993)

### Problem formulation

#### **Definition 1**

(*Electronic Health Record | EHR*) EHR is the hierarchical co-occurrence time sequence data. It consists of a set of records $R = \left\{ {r_{i} |i = 1, \ldots ,M} \right\}$ with $M$ patients $P$. Each record $r_{i} { }$ has a visit sequence $V = \left\{ {v_{i} |i = 1, \ldots ,N} \right\}$ mapped in time. For each $v_{i}$, it contains a time stamp $t_{i}$ and many codes $c_{i} = \{ c_{ij} | c_{ij} \varepsilon C,j = 1, \ldots ,J\}$. $C$ is a diagnoses database. Meanwhile, the demographic information $I$ is recorded to ${\text{patients }}P$.

#### **Definition 2**

(*Medical prediction tasks*) They use a set of medical records $R$ to predict the specific target $Y = \left\{ {y_{1} ,y_{2} , \ldots y_{n} } \right\}$. If $n = 2$, it is a two-classification task. If $n > 2$, it is a multi-classification task. The prediction task is $f_{p} :R \to Y$.

#### **Definition 3**

(*Interpretation Path*) Interpretation uses the correlations ${\mathcal{R}}$ of medical pairs $Q$ to build an Interpretation Path ${\mathcal{P}}$. $Q$ is a set of tuple $\left\{ {\left( {a,b} \right)|a\varepsilon C \cup V, b\varepsilon C \cup V \cup P \cup Y} \right\}$, the pair correlation is $a{\mathcal{R}}b$, and ${\mathcal{P}} = c_{1} \to ^{{ {\mathcal{R}}_{1} }} c_{2} \to ^{{ {\mathcal{R}}_{2} }} \ldots \to ^{{ {\mathcal{R}}_{n - 1} }} c_{n} \to ^{{ {\mathcal{R}}_{n} }} prediction$ interprets how TCoN predicts.

### Analysis strategy

**Task 1** (Mortality prediction). To predict if the patient will die during the hospitalization.

**Task 2** (Readmission prediction). To predict if the patient will be hospitalized again.

**Task 3** (Disease prediction). Two disease prediction tasks: Sepsis and heart failure. Early diagnose is critical for improving patients’ outcome [35].

**Task 4** (Next diagnoses prediction). To predict the diagnoses of the patient in the next admission.

Note that Task 1, 2, 3 are binary classification tasks and Task 3 is a multi-classification task.

**Evaluation 1** (AUC-ROC). The area under the curve of the True Positive Rate (TPR) and the False Positive Rate (FPR). TN, TP, FP, and FN represent true positives, true negatives, false positives, and false negatives, respectively.

1 $$\begin{aligned} & TPR = \frac{TP}{{TP + FN}} \\ & FPR = \frac{FP}{{TN + FP}} \\ \end{aligned}$$

**Evaluation 2** (PR-AUC). The area under the curve of Precision (P) and Recall (R). It is a better measure for imbalanced data [36].

2 $$\begin{aligned} & P = \frac{TP}{{TP + FP}} \\ & R = \frac{TP}{{TP + FN}} \\ \end{aligned}$$

**Evaluation 3** (Accuracy@k). The probability of the positive predictions in top-k prediction values. It is the evaluation metric of multi-classification tasks.

3 $$Accuracy@k = { }\frac{\# \;of\;true\;positive\;in\;top\;k}{{{\text{min}}\left( {k,\left| {c_{t} } \right|} \right)}}$$

### TCoN model structure

**Fig. 2:**
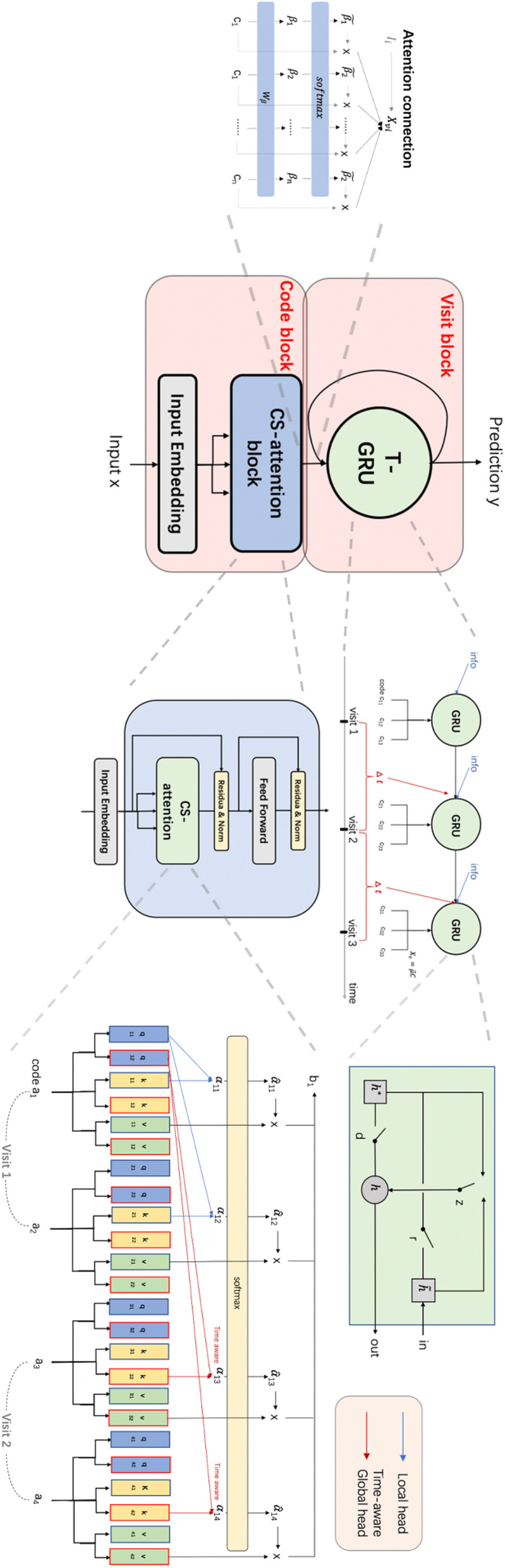
TCoN structure

As shown in Fig. 2, our TCoN model contains the code block and the visit block: The code block is implemented by Co-occurrence-aware Self-attention (CS-attention); The visit block is implemented by Time-aware Gated Recurrent Unit (T-GRU); Two blocks are connected by Attention connection.

#### CS-attention

Self-attention [32] in natural language processing considers the semantic and grammatical relations between different words in sentences. For each input, it has three vectors, Query (Q), Key (K), and Value (V). The multi-head self-attention is designed as:

4 $$\begin{aligned} & Attention\left( {Q,{ }K,{ }V} \right) = softmax\left( {\frac{{QK^{T} }}{{\sqrt {D_{k} } }}} \right)V \\ & MultiHead\left( {Q,{ }K,{ }V} \right) = Concat\left( {head_{1} , \ldots ,head_{h} } \right)W^{O} \\ & head_{i} = Attention\left( {QW_{i}^{Q} ,{ }KW_{i}^{K} ,{ }VW_{i}^{V} } \right) \\ \end{aligned}$$

In this work, we redesign the self-attention as CS-attention (Eq. 5) to deal with the relations of EHR codes. CS-attention has two different heads—Local Head and Global Head. The local head learns the co-occurrence relations between every two codes in the same visit. A code is affected by the other codes equally. The global head learns the co-occurrence relations between every two codes in different visits. A code has different effects from the other codes according to different time intervals between visits. These two types of heads can learn a new representation $\tilde{C }$ of each code $C$ by its neighbors ${C}_{nb}$.

5 $$\tilde{C} = Attention\left( C \right) = softmax\left( {\frac{{Q_{1} K_{1}^{T} }}{{\sqrt {d_{k} } }},\frac{{ (Q_{2} K_{2}^{T} )^{T} T}}{{\sqrt {d_{k} } }}} \right)\left( {C,C_{nb} } \right)$$

$C$ is the original matrix of input codes. $\tilde{C}$ is the new representation matrix. $Q_{i} ,K_{i} , V_{i}$ is same as Eq. (4). $i = 1$ represents the local head and $i = 2$ represents the global head. $d_{k}$ is the dimension for $Q$ and $K$. $T$ is the time decay function $g\left( {\Delta t} \right)$ in Eq. 4. Both the number of local head and global head can be change.

#### T-GRU

As shown in Eq. 3, T-GRU comprises an update gate ${z}_{t}$ and a reset gate ${r}_{t}$. They control the extent to which the previous state ${h}_{t-1}$ is brought into the current state ${h}_{t}$ and how far the previous state is brought into the current candidate state ${\tilde{h }}_{t}$. For modeling the time irregularity, we build a time gate ${d}_{t}$. This gate takes time interval into account and control delivered information from the previous visit to the current visit by time decay function $g\left( {\Delta t} \right)$. The time decay function can determine how much the history state can be injected into the current unit. In Eq. 3, ${x}_{t}$ is the current input data, $W, U, b$ are parameters. The output is the current state $h$.

6 $$\begin{aligned} & {\text{Time}}\;{\text{gate}}:\;\;d_{t} = g\left( {\Delta t} \right) \\ & {\text{Time}}\;{\text{decayed}}\;{\text{history}}\;{\text{state}}\quad h_{t - 1}^{*} :h_{t - 1}^{*} = h_{t - 1} \cdot {d_{t}} \\ & {\text{Update}}\;{\text{gate}}\;\;z_{t} :\;\;z_{t} = \sigma \left( {x_{t} \cdot {W_{z}} + h_{t - 1}^{*} \cdot {U_{z}} + b_{z} } \right) \\ & {\text{Reset}}\;{\text{gate}}\;\;r_{t} :\;\;r_{t} = \sigma \left( {x_{t} \cdot {W_{r}} + h_{t - 1}^{*} \cdot {U_{r}} + b_{r} } \right) \\ & {\text{Candidate}}\;{\text{state}}\;\;\tilde{h}_{t} :\;\;\tilde{h}_{t} = \left( {x_{t} \cdot {W_{h}} + h_{t - 1}^{*} \cdot {r_{t}} \cdot U_{z} + b_{h} } \right) \\ & {\text{Current}}\;{\text{state}}\;\;h_{t} :\;\;h_{t} = z_{t} \cdot h_{t - 1}^{*T} + \left( {1 - z_{t} } \right) \cdot \tilde{h}_{t} \\ \end{aligned}$$

We propose three time decay functions (Eq. 7). Δ*t* is the time interval between two visits, $\alpha$ is the decay rate. When $\alpha =1$, the exponential form is more suitable for the small elapsed time, the logarithmic form is more suitable for the large elapsed time, and the reciprocal form is a compromise.

7 $$\begin{aligned} & {\text{Reciprocal}}\;{\text{form}}\quad g\left( {\Delta t} \right) = \frac{1}{1 + \alpha \Delta t} \\ & {\text{Logarithmic}}\;{\text{form}}\quad g\left( {\Delta t} \right) = \frac{1}{{{\text{log}}\left( {e + \alpha \Delta t} \right)}} \\ & {\text{Exponential}}\;{\text{form}}\quad g\left( {\Delta t} \right) = e^{ - \alpha \Delta t} \\ \end{aligned}$$

#### Attention connection

Between code block and visit block, we design the connection method (Eq. 8). Where $X_{vi}$ is the *i*th input of visit $v$, $C_{i}$ is the output matrix with each row for one $i$-th visit’s code, $W_{\beta }$ is a parameter vector. When we consider the demographic information $I$. The input will be a concatenation form: $X_{vi} = concate\left( {\tilde{\beta }^{T} C_{i} , I_{i} } \right)$.

8 $$\begin{aligned} & \beta = C_{i} W_{\beta } + b_{\beta } \\ & \tilde{\beta } = softmax\left( \beta \right) \\ & X_{vi} = \tilde{\beta }^{T} C_{i} \\ \end{aligned}$$

Besides, we propose a method to interpret TCoN. It is achieved by the correlation values among codes, visits, and predictions.

#### Interpretation path

It is based on the correlations $\mathcal{R}$, containing two correlations: The code-code correlation is obtained from $\widehat{\alpha }$ of CS-attention. ${\widehat{\alpha }}_{ij}$ means the effect of code $j$ on code $i$, and large ${\widehat{\alpha }}_{ij}$ means that code $j$ could be the cause, complication, or early symptoms of code $i$; The code-visit correlation is obtained from $\stackrel{\sim }{\beta }$ of the Attention connection. Larger $\stackrel{\sim }{\beta }$ means the closer relation.

The interpretation path is a code sequence obtained by the reverse lookup starting with the prediction results. For a prediction $P$, the last visit is ${v}_{n}$. In ${v}_{n}$, we find the code ${c}_{ni}$ that contributed the most to ${v}_{n}$ according to $\stackrel{\sim }{\beta }$. For ${c}_{ni}$, we find the closest code ${c}_{(n-1)i}$ in visit ${v}_{n-1}$ according to the largest ${\widehat{\alpha }}_{{*C}_{ni}}$. Similarly, we find ${c}_{(n-2)i},{c}_{(n-3)i},\dots {c}_{1i}$. So far, we find a path ${c}_{1i}\to {\dots \to c}_{ni}\to P$. This path can be described: a disease ${c}_{1i}$ most likely infers ${c}_{2i}$, then ${c}_{2i}$ most likely infers ${c}_{3i}$, … and ${c}_{(n-1)i}$ most likely infers ${c}_{ni}$, finally, ${c}_{ni}$ most likely causes $P$.

Finally, we apply a training method that enables TCoN to handle imbalanced data [37, 38].

#### Pre-train and Fine-tune

In the pre-train process, we apply an auto-encoder network ${f}_{ae}$ with a minimum loss (Eq. 9) for the unsupervised representation learning task. In the fine-tune process, we use parameters of the encoder layer as the initial parameters of TCoN when training by the prediction objective in Eq. (10). For TCoN, the input layer is represented by Eq. (8), Skip-connection is Eq. (12), layer normalization [29] is Eq. (13), and feed forward layer is Eq. (14).

9 $$L_{emb} = - \frac{1}{n}\mathop \sum \limits_{i}^{n} x_{i} logf_{ae} \left( {x_{i} } \right)$$

10 $$L_{pre} = - \frac{1}{n}\mathop \sum \limits_{i}^{n} y_{i} logf_{pre} \left( {x_{i} } \right)$$

11 $$a = emb\left( x \right) = ReLU\left( {x \cdot W + b} \right)$$

12 $$x^{\prime} = RC\left( x \right) = x + f\left( x \right)$$

13 $$x^{\prime} = \gamma \frac{x - \mu }{{\sqrt {\sigma + \varepsilon } }} + \beta$$

14 $$c = FF\left( b \right) = Relu\left( {b \cdot W_{1} + b_{1} } \right) \cdot W_{2} + b_{2}$$

### Complexity analysis

The self-attention-based algorithm is parallel, but the RNN-based algorithm is serial [32]. TCoN has both structures and they are connected in series. Thus, the complexity of TCoN is $O\left({n}^{2}\cdot d\right)=O\left({n}^{2}\cdot d\right)+O\left(n\cdot {d}^{2}\right)$. $d$ is the representation dimension and $n$ is the sequence length. $O\left({n}^{2}\cdot d\right)$ is the complex of CS-attention with ${n}^{2}$ for operations of every two inputs. $O\left(n\cdot {d}^{2}\right)$ is the complex of T-GRU with ${d}^{2}$ for sequential operation. In our data, the dimensionality $d$ is smaller than the data length $n$, so that the complex of TCoN is $O\left(n\cdot {d}^{2}\right)$.

## Results

### Experimental setup

For data, we right align the time series and use padding and masking to make them equal in length. Each code is represented by a one-hot vector with 4,893 dimensions (number of ICD-9 codes). Training, validation, and testing set is in 0.75:0.1:0.15 ratio.

For model, we set 2 local heads and 2 global heads. We choose $\alpha =1$ logarithmic time decay with year as the decay unit. We apply Adam Optimizer [39] with $\alpha =0.001$, ${\beta }_{1}=0.9$ and ${\beta }_{2}=0.999$. We use the learning rate decay method ${\alpha }_{current}=$ ${\alpha }_{initial}\cdot {\gamma }^{\frac{global\; step}{decay\; steps}}$ with decay rate $\gamma =0.98$ and decay step = 2000 [40]. Before the prediction task, we carry out the pre-train step and use the early stop with 5 epochs. We use the fivefold cross-validation. The code implementation is publicly available at https://github.com/SCXsunchenxi/MTGRU

### Baselines

- Time-aware methods (RNN-based methods)
  - GRU [14]. It uses GRU to embed visits and make the final prediction.
  - T-LSTM [8]. It uses elapsed time weight to change previous memory in LSTM.
- Co-occurrence-aware methods (Word2Vec-based methods)
  - Med2Vec [4]. It applies the skip-gram model and multi-layer perceptron to get the representation of codes and visits.
  - Dipole [1]. It uses BRNN along with three attention mechanisms to measure the relation of different visits for the final prediction.

### Prediction results

TCoN predicts more accurately than all baselines. The results of binary classification (mortality, readmission, sepsis, and heart failure) and multi-classification (next diagnoses) are shown in Table 2(a, b). Baselines may not match EHR characteristics and partially model data features. For example, T-LSTM has the worst performance as it is not suitable for short visit sequences like MIMIC-III.

**Table 2:** Prediction results of mortality, readmission, sepsis, heart failure and next diagnoses

Method	Mortality		Readmission		Sepsis		Heart Failure	
	ROC-AUC	PR-AUC	ROC-AUC	PR-AUC	ROC-AUC	PR-AUC	ROC-AUC	PR-AUC
(a) *Results of binary classification prediction*								
GRU	0.7902	0.7400	0.7023	0.6713	0.6202	0.6063	0.6525	0.6187
Med2Vec	0.8025	0.7950	0.7125	0.6833	0.8211	0.7943	0.7225	0.7101
Dipole	0.8133	0.8103	0.7341	0.7243	0.8001	0.7823	0.7067	0.6923
TLSTM	0.7893	0.7392	0.7256	0.7023	0.6432	0.6189	0.7432	0.6033
TCoN	0.8224	0.8134	0.7403	0.7278	0.8433	0.8233	0.7698	0.7313

	Accuracy@5	Accuracy@15	Accuracy@25	Accuracy@35
(b) *Multi-classification result of next diagnoses prediction*				
GRU	0.7723	0.6298	0.5801	0.4523
Med2Vec	0.8025	0.7061	0.6250	0.5025
Dipole	0.8043	0.6514	0.6012	0.5044
TLSTM	0.7833	0.6367	0.5814	0.4515
TCoN	0.8398	0.7223	0.6577	0.5113

TCoN performs well on imbalanced datasets. In binary classification tasks, all datasets are imbalanced, especially the sepsis dataset (6.16%). But the results show that the more imbalanced the data, the greater the advantage of TCoN over baselines.

TCoN can accurately predict multiple diagnoses in the next admission. In the multi-classification task, we evaluate methods with $\mathrm{k}$ = 5, 15, 25, 35. As shown in Table 2b, as $\mathrm{k}$ increases, the accuracies of all methods decrease, but the advantage of our approach is still obvious.

### Model parameters experiments

**Fig. 3:**
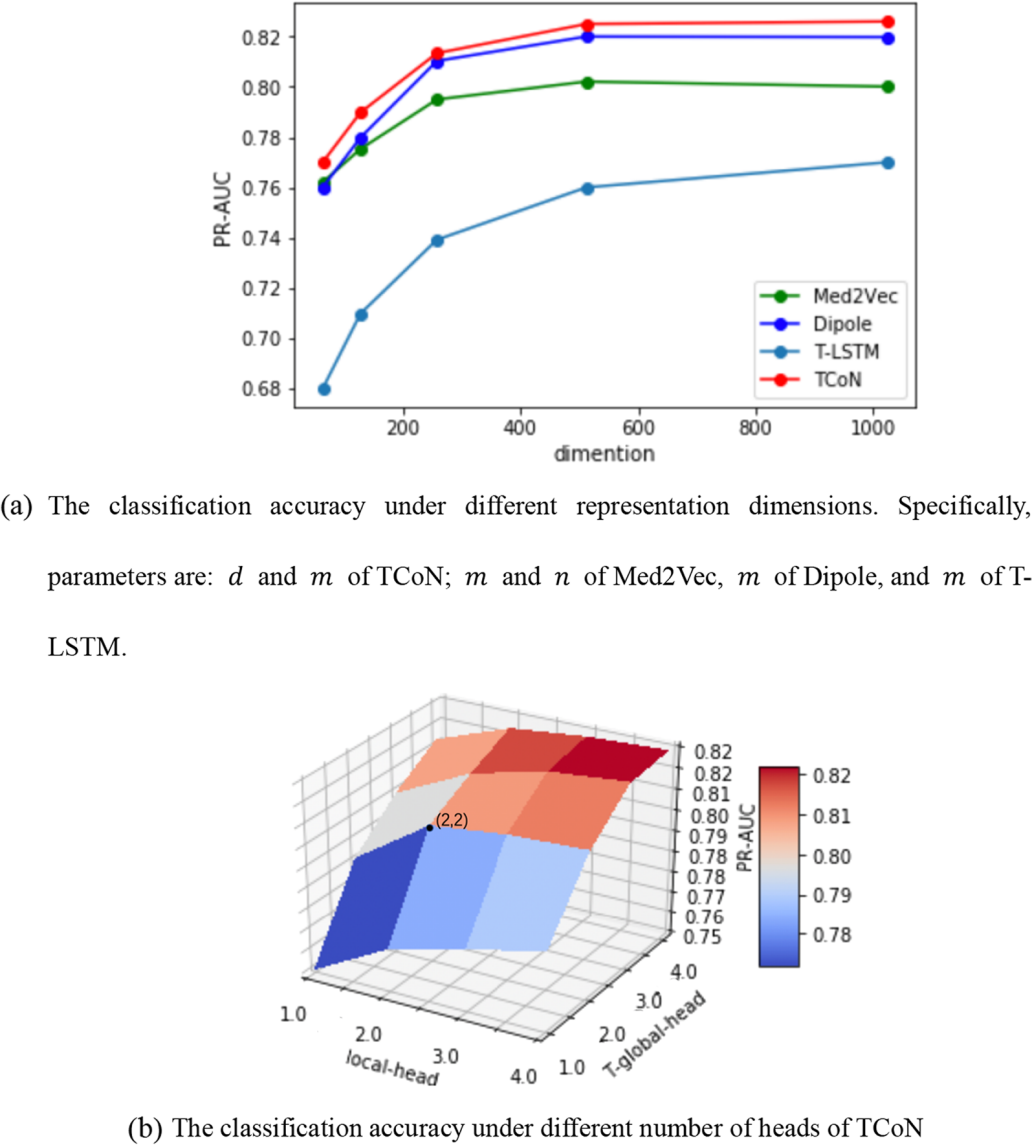
The classification accuracy under different model parameters

We change the dimension of representation vector in hidden layers. The results in Fig. 3a show that TCoN performs better than other methods under all dimensions. Then, we set different numbers of heads for TCoN. Figure 3b shows that the number of heads = 2 is the key turning point.

### Case study of interpretation path

**Fig. 4:**
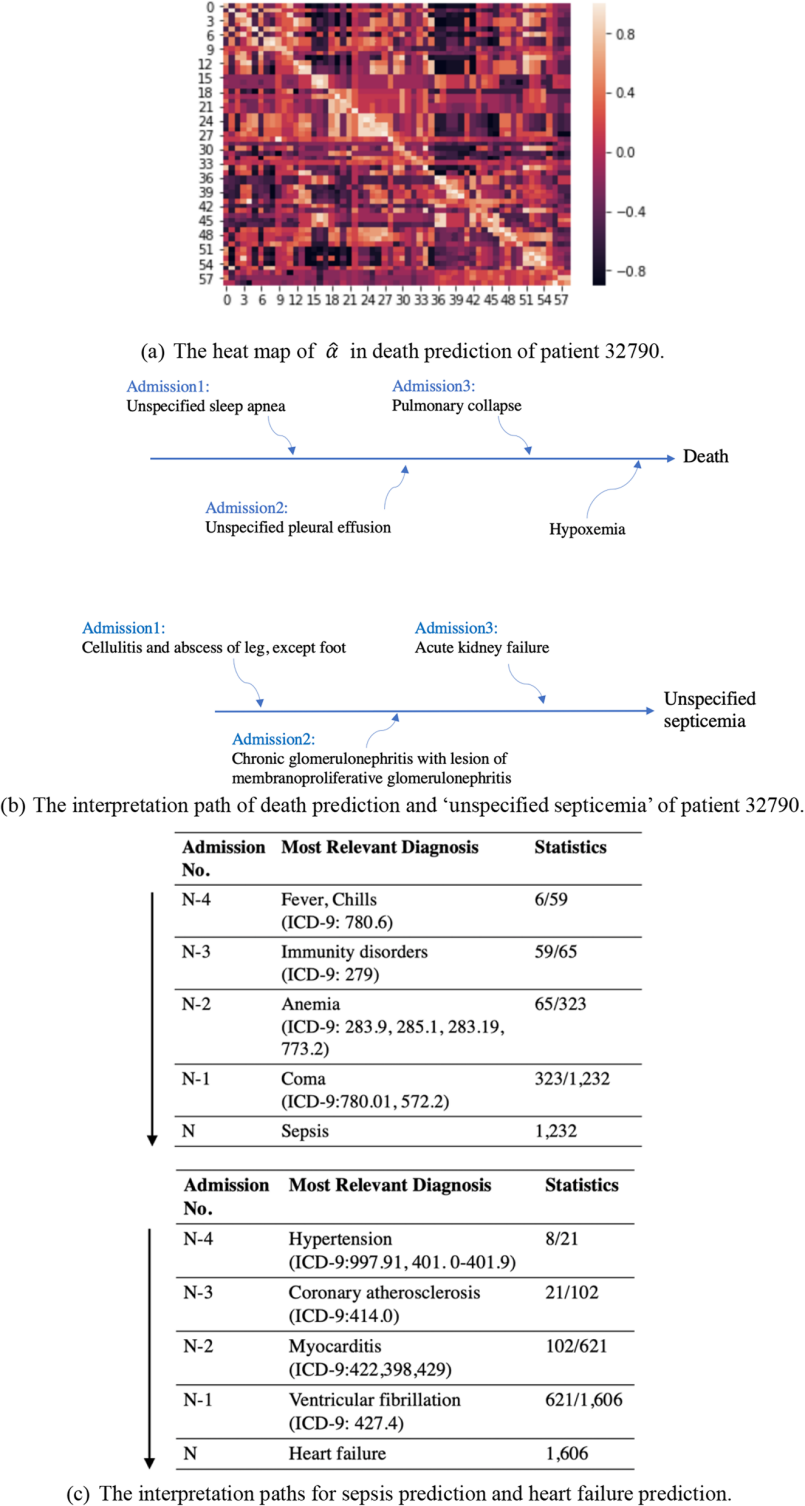
Interpretation path

We choose a patient numbered 32,790 in MIMIC-III (a white man with 3 admission records and died at 80) to describe how TCoN produces the interpretation path. Figure 4a is the heat map of $\widehat{\alpha }$ for the death prediction. The diagnosis ‘hypoxemia’ contributes the most to the last admission as its weighted vector’s norm is the biggest. For ‘hypoxemia’, the closest diagnosis is ‘pulmonary collapse’ with the biggest ${\widehat{\alpha }}_{*i}=0.892$. For ‘pulmonary collapse’, the closest diagnosis is ‘unspecified pleural effusion’ with the biggest ${\widehat{\alpha }}_{*i}=0.803$. And for ‘unspecified pleural effusion’, the closest diagnosis is ‘unspecified sleep apnea’ with the biggest ${\widehat{\alpha }}_{*i}=0.782$. So far, an interpretation path ‘unspecified sleep apnea —>  unspecified pleural effusion —> pulmonary collapse —>  Hypoxemia —>  death’ is found as shown in Fig. 4b.

Figure 4c shows cases of interpretation paths of sepsis prediction and heart failure prediction. Each path is the summary results by using the most frequent diagnosis. Thus, we find sepsis-related pre-diagnoses/symptoms, such as ‘Fever’, ‘Chills’, ‘Immunity disorders’, ‘Anemia’ and ‘Coma’. And we find heart failure-related pre-diagnoses/symptoms, such as ‘Ventricular fibrillation’, ‘Myocarditis’, ‘Coronary atherosclerosis’ and ‘Hypertension’.

## Discussion

In recent years, deep learning (DL) technology has shown its superior performance in medical applications [41–44], such as medical image recognition [45] and medication recommendations [46]. And many methods have achieved good performance for specific disease prediction, such as Alzheimer's disease [47], sepsis [48], and heart disease [49, 50]. However, most of them pursue the task accuracy but ignoring the interpretability. DL-based approaches are black-box models, which is not easy to understand for non-professionals, especially doctors without artificial intelligence backgrounds. Thus, the explainable DL method is needed. This study aims at this problem and puts forward a solution, interpretation path, to make the predictions explainable.

In EHR, the patient's records are irregular in time due to the unpredictability of the diseases and inevitable data loss. The current disease could be more closely related to the disease a week ago than the disease a year ago [8, 9]. Thus, the time perception mechanism is needed. This study aims at this issue and proposes a time gate to explicitly learn the irregular time information by the time decay function.

**Fig. 5:**
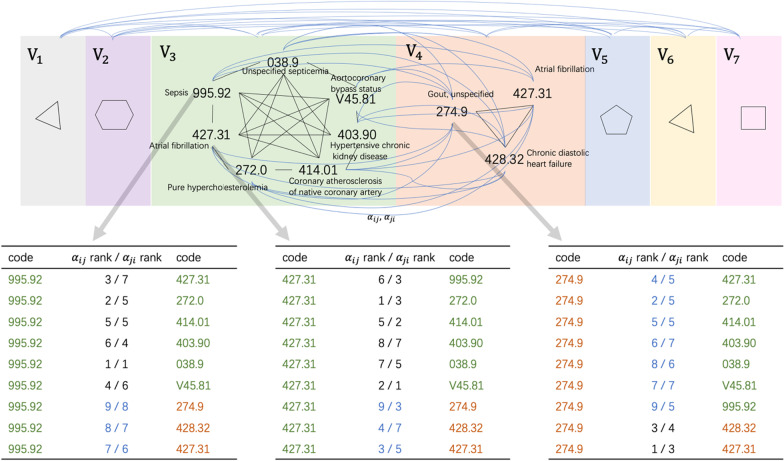
Diagnosis graph of patient 32,790. Patient 32,790 has 7 visits, shown as 7 blocks with different colors. For clarity, we only show the ICD-9 code of diagnoses in visit 3, 4. The black line represents the code relations in the same visit, the blue lines represent the code relations in different visits. The relation closeness is measured by the edge weight. Every edge between code $i$ and $j$ has two weights ${\alpha }_{ij}$ and ${\alpha }_{ji}$. Three tables record the relations between code 995.52 (sepsis), code 427.31 (atrial fibrillation), code 274.9 (gout) with other codes

The experiments show that using two kinds of head for relations of inter-visit and intra-visit is necessary. The difference between these two relations is not just the time interval, but also the pathology. We emphasize the code relations are more likely to be complications in the same visit, but causations and continuities among different visits. For example, in our experiments, the relation of ‘diabetes’ with ‘cellulitis and abscess of legs’ in one visit is more prone to be a short-term complication, but the relation of ‘diabetes’ and ‘long-term use of insulin’ in two different visits is more prone to be causation. Thus, for each patient, we can give a disease association graph. The weight of the edges between two diagnoses in the same admission represents the adjoint coefficient, and the weight of the edges between two diagnoses in different admissions represents the causal coefficient. Figure 5 shows the diagnosis graph case of patient 32,790.

The interpretation path is not symmetrical, which means ${\widehat{\alpha }}_{ij}\ne {\widehat{\alpha }}_{ji}$. ${\widehat{\alpha }}_{ij}$=$\frac{\#\, of\, i-j\, occurrences}{\# of \, i\, occurrences}$ and ${\widehat{\alpha }}_{ji}= \frac{\#\, of\, i-j\, occurrences}{\#\, of\, j\, occurrences}$, they have different denominators. For example, code $i$, $j$, $k$ represent the diagnoses of ‘malaria’, ‘fever’, ‘periodic cold fever’ respectively. In our experiment, $i$ is mostly accompanied by $j$ as ${\widehat{\alpha }}_{ij}=0.762$. But $j$ is not always accompanied by $i$ as ${\widehat{\alpha }}_{ji}=0.023$. It is mostly accompanied by code $k$ with ${\widehat{\alpha }}_{ki}=0.701$. Comparing ${\widehat{\alpha }}_{ji}$ and ${\widehat{\alpha }}_{ki}$, the results show that ‘periodic cold fever’ is a better explanation for ‘malaria’ than ‘fever’. In research [51], ‘periodic cold fever’ is a special clinical manifestation of ‘malaria’ and there are very few other diseases with this symptom. It illustrates that our interpretable method can explain the results by reflecting the relation (such as complication, causation, and continuity) between the diagnoses and ${\widehat{\alpha }}_{*i}$ is a more important standard to find the maximum co-occurrence code for $i$ than ${\widehat{\alpha }}_{i*}$.

In medical applications, the data is usually imbalanced. The normal state of patients is the majority, while the disease records may be the small sample. But the small sample is more important for the disease prediction. Thus, a DL model should be robust on the imbalanced dataset. In this paper, our pre-train and fine-tune framework can help.

Further, there is room for further improvement. The current modeling method is based on pure EHRs data. Integrating prior information will make the results of the data relation modeling and medical prediction more accurate and reasonable. The available method is knowledge graph embedding based on ICD code. Besides, more data in EHRs such as doctor notes, medications, and laboratory tests can be used for better performance. Future work will focus on these aspects.

## Conclusion

The data-driven medical prediction method based on interpretable deep learning is essential for healthcare management. In this paper, we propose an interpretable Time-aware and Co-occurrence-aware Network (TCoN) for data modeling and medical prediction. It can perceive hierarchical data structures with the time relation and the co-occurrence relation, give an interpretation path to explain the prediction, and build a diagnosis graph for every patient. The experiments show that TCoN outperforms the state-of-the-art methods.

## Data Availability

The code implementation is publicly available at https://github.com/SCXsunchenxi/MTGRU. The data is at https://mimic.physionet.org.

## Abbreviations

EHR: Electronic health record
ICD: International classification of diseases
WHO: World Health Organization
DL: Deep learning
RL: Representation learning
RNN: Recurrent neural network
CNN: Convolutional neural network
BRNN: Bidirectional recurrent neural network
LSTM: Long short-term memory
GRU: Gated recurrent unit
TCoN: Time-aware and co-occurrence-aware deep learning network
CS-attention: Co-occurrence-aware self-attention
T-GRU: Time-aware gated recurrent unit
AUC-ROC: The area under the curve of receiver operating characteristic
PR-AUC: The area under curve of precision-recall
TP: True positives
TN: True negatives
FP: False positives
FN: False negatives
TPR: True positive rate
FPR: False positive rate
